# Syphilitic Hepatitis: An Uncommon Manifestation of Secondary Syphilis

**DOI:** 10.7759/cureus.74524

**Published:** 2024-11-26

**Authors:** Kimberly J Richardson, George C Yen

**Affiliations:** 1 Department of Medicine, David Geffen School of Medicine, University of California, Los Angeles (UCLA) Health, Los Angeles, USA

**Keywords:** hepatitis, rash, syphilis, transaminitis, treponema

## Abstract

Syphilitic hepatitis is a rare manifestation of a sexually transmitted infection. Given its nonspecific presentation, it is important for clinicians to consider the diagnosis in sexually active patients presenting with elevated liver tests.

In this case, a 30-year-old man presented with an itchy rash and was diagnosed with an allergic reaction. He returned to the clinic one week later with a worsening rash. A sexual history was obtained, which led to sexually transmitted disease testing. Laboratory evaluation revealed a positive syphilis serology. A diagnosis of secondary syphilis was made, and he was treated with penicillin. The following day, he presented to the emergency department complaining of worsening rash, abdominal pain, and fever. Laboratory evaluation was significant for elevated transaminases. He received supportive care and was advised to follow up with his primary care provider. One month after his initial presentation, he presented to the clinic with resolution of the rash. Laboratory evaluation revealed a fourfold decrease in rapid plasma reagin titer and improvement in liver enzymes. The transaminitis work-up was unremarkable, and the liver tests ultimately normalized. A diagnosis of syphilitic hepatitis was made. A comprehensive detailed sexual history is important and key to the diagnosis of syphilitic hepatitis. Long-term sequelae are rare. Syphilitic hepatitis is a curable condition and should remain in the differential diagnosis in all sexually active persons presenting with abnormal liver enzymes.

## Introduction

Syphilis is a sexually transmitted systemic bacterial infection caused by the spirochete *Treponema pallidum*. It remains a significant public health issue, especially among men who have sex with men (MSM) [1]. Syphilis progresses through stages including primary, secondary, latent, and tertiary without treatment. The chancre lesion is seen in the primary stage and commonly involves the genitals [1,2]. The secondary stage involves the dissemination of the treponemes through the bloodstream resulting in fever, rash, and adenopathy [1]. The latent stage has no visible signs or symptoms, and the tertiary stage is characterized by neurosyphilis, cardiovascular effects, and gumma formation [2]. Syphilis cases are on the rise. Here, we present a case of secondary syphilis.

## Case presentation

A 30-year-old man with a past medical history significant for human immunodeficiency virus (HIV), well-controlled on bictegravir-emtricitabine-tenofovir, presented to an immediate care center complaining of an itchy rash involving the scalp, face, neck, chest, and abdomen for the past four days. He denied recent exposures including topicals, oral medications, or new food items. There was no associated fever, chills, headache, nausea, vomiting, diarrhea, dysuria, dizziness, lightheadedness, chest pain, shortness of breath, wheezing, or cough. Vital signs were as follows: blood pressure 133/73, pulse rate 79, temperature 36.5°C, respiratory rate 16, saturation of peripheral oxygen (SpO2) 96%, height 6’3”, and weight 240 lbs. The physical exam was remarkable for a diffuse erythematous maculopapular rash. There were no bullae, vesicles, clustering of lesions, pus, or exudates present. There was no involvement of the palms or soles. He was diagnosed with an allergic reaction and treated with oral prednisone, hydroxyzine, and topical triamcinolone.

He returned to the immediate care center one week later stating that the rash improved initially and then worsened. He endorsed condomless sex with a new partner one month prior and requested testing for sexually transmitted infections. The vital signs were stable and the physical exam was unchanged. Laboratory evaluation was significant for a reactive rapid plasma reagin (RPR) with reflex titer ≥1:1024. He was diagnosed with secondary syphilis and treated with one dose of intramuscular penicillin G benzathine 2.4 million units.

He presented to the emergency department the following day complaining of a diffuse rash associated with intense itching, inability to sleep, abdominal pain, and subjective fever consistent with a Jarisch-Herxheimer reaction. His review of systems was otherwise negative. Vital signs were as follows: blood pressure 142/82, pulse rate 81, temperature 36.5°C, respiratory rate 17, SpO2 98%, height 6’3”, and weight 240 lbs. Physical exam was remarkable for a diffuse erythematous maculopapular rash and soft abdomen without tenderness, guarding, or rebound. Laboratory evaluation was significant for alkaline phosphatase 240 U/L, aspartate aminotransferase 310 U/L, and alanine aminotransferase 539 U/L (Table 1). He was discharged home on diphenhydramine and ibuprofen for symptom relief and advised to follow up with his primary care provider for further evaluation and management.

**Table 1:** Patient test results RPR: rapid plasma reagin; TP-EIA: *Treponema pallidum* enzyme immunoassay; ALP: alkaline phosphatase; AST: aspartate aminotransferase; ALT: alanine aminotransferase

Laboratory test	Pretreatment	Posttreatment	Reference range
RPR	≥1:1024	≥1:256	Nonreactive
TP-EIA	Reactive	Reactive	Nonreactive
ALP	240 U/L	96 U/L	37-113 U/L
AST	310 U/L	199 U/L	13-62 U/L
ALT	539 U/L	136 U/L	8-70 U/L

The patient followed up in the clinic one month after his initial presentation, and the rash had resolved. He denied new medications, herbal supplements, or alcohol or substance use. The repeat RPR was reactive with reflex titer 1:256. The follow-up comprehensive metabolic panel was significant for alkaline phosphatase 96 U/L, aspartate aminotransferase 199 U/L, and alanine aminotransferase 136 U/L (Table 1), all of which were improving. Further evaluation of the transaminitis including acute hepatitis panel, ceruloplasmin, alpha-1-antitrypsin, serum immunoglobulin G (IgG), liver kidney microsome antibody, smooth muscle antibody, celiac antibody, antinuclear antibody, and iron studies was unremarkable. Three months later, a repeat RPR with a reflex titer was 1:2, and the patient's liver enzymes returned to normal. His clinical history and laboratory findings were consistent with a diagnosis of syphilitic hepatitis. 

## Discussion

Syphilitic hepatitis most commonly occurs in the secondary stage of syphilis. However, this is a rare complication, as it is estimated that only 3% of secondary syphilis cases will present with hepatitis [3]. Clinical manifestations include rash, fatigue, poor appetite, scleral icterus, uveitis, fever, weight loss, abdominal pain, arthralgia, lymphadenopathy, splenomegaly, phallodynia, sore throat, and/or headache [3,4]. Although there are no well-defined diagnostic criteria, syphilitic hepatitis can be reasonably diagnosed without pathology when the following criteria are met: elevated liver enzymes including elevations in alkaline phosphatase, positive syphilis serology with a non-treponemal test such as the RPR, exclusion of other etiologies of liver disease, and resolution of transaminitis following appropriate antimicrobial therapy [3,4]. If obtained, histologic confirmation can show lymphoplasmacytic infiltrates, granulomas, and bile duct damage, but these findings are not specific to syphilis [3,4]. Most cases resolve after treatment, and fulminant hepatitis is rare [3].

Syphilitic hepatitis can be overlooked because presenting symptoms are usually nonspecific [2]. The rash is often nonpruritic, erythematous, and maculopapular concentrated on the trunk, palms, and soles [2]. This patient's rash was pruritic and did not involve the palms or soles leading to an initial misdiagnosis as an allergic reaction. Laboratory evaluation may show an increase in alkaline phosphatase with elevation in alanine aminotransferase or aspartate aminotransferase levels [2]. This patient did not require a liver biopsy, as his liver enzymes improved significantly with antimicrobial therapy. Studies show that syphilitic hepatitis is more common in men living with HIV, specifically MSM, which may be related to the route of sexual transmission [2].

The prevalence of syphilis has been rising in recent years [1,5]. The screening, prevention, and treatment of syphilis should be integrated into primary care for all sexually active people including those living with HIV. Doxycycline post-exposure prophylaxis (Doxy-PEP) is a new pharmacologic tool that can reduce the incidence of syphilis. A recent study showed that 200 mg of doxycycline, if taken within 72 hours of condomless sex, reduced gonorrhea, chlamydia, and early syphilis by two-thirds in MSM and transgender women who have had a recent history of a bacterial sexually transmitted infection within the past 12 months [6]. 

## Conclusions

Syphilitic hepatitis is a rare manifestation of secondary syphilis that may be missed due to its nonspecific presentation. Given the rising incidence of syphilis, clinicians should have a high index of suspicion for syphilitic hepatitis when a sexually active patient presents with rash and elevated liver enzymes, particularly a rise in alkaline phosphatase. Penicillin remains the first-line treatment for all stages of syphilis. For syphilitic hepatitis, treatment response is defined as a fourfold decrease in RPR titers and resolution of transaminitis.
